# Sex and Gender Interactions on the Use and Impact of Recreational Cannabis

**DOI:** 10.3390/ijerph17020509

**Published:** 2020-01-14

**Authors:** Lorraine Greaves, Natalie Hemsing

**Affiliations:** 1 Centre of Excellence for Women’s Health, Vancouver, BC V6H 3N1, Canada; lgreaves@cw.bc.ca; 2 School of Population and Public Health, University of British Columbia, Vancouver, BC V6T 1Z4, Canada

**Keywords:** sex, gender, cannabis

## Abstract

Cannabis is the second most frequently used substance in the world and regulated or legalized for recreational use in Canada and fourteen US states and territories. As with all substances, a wide range of sex and gender related factors have an influence on how substances are consumed, their physical, mental and social impacts, and how men and women respond to treatment, health promotion, and policies. Given the widespread use of cannabis, and in the context of its increasing regulation, it is important to better understand the sex and gender related factors associated with recreational cannabis use in order to make more precise clinical, programming, and policy decisions. However, sex and gender related factors include a wide variety of processes, features and influences that are rarely fully considered in research. This article explores myriad features of both sex and gender as concepts, illustrates their impact on cannabis use, and focuses on the interactions of sex and gender that affect three main areas of public interest: the development of cannabis use dependence, the impact on various routes of administration (ROA), and the impact on impaired driving. We draw on two separate scoping reviews to examine available evidence in regard to these issues. These three examples are described and illustrate the need for more comprehensive and precise integration of sex and gender in substance use research, as well as serious consideration of the results of doing so, when addressing a major public health issue such as recreational cannabis use.

## 1. Introduction

Cannabis is the second most frequently used substance in the world, after alcohol [1]. It is an illegal substance in most countries, but increasingly becoming regarded as a controlled substance in various states in the USA and, as of 2018, all of Canada [2]. In 2018, recreational cannabis was legalized in Canada, 17 years after the regulation of medical use. Recreational cannabis use is also legal in Uruguay–for personal use since 1974, and for cultivation and sale since 2013 [3]. Eleven US states plus Washington, D.C. and two US territories (Guam, Northern Mariana Islands) have also introduced legal recreational cannabis use among adults and fifteen US states have decriminalized cannabis. Cannabis is semi-legal in several other countries. For example, Argentina, South Africa, and Mexico have identified punishment for possession of cannabis for personal consumption as unconstitutional; the Netherlands tolerates public consumption and sale of cannabis in licensed coffee-shops; and in Spain personal consumption and cultivation of cannabis is tolerated [3].

When legalization of recreational cannabis occurred in Canada in October 2018, efforts to research the impacts of legalization and use were accelerated and numerous key clinical and public policy issues emerged. In Canada, 17.1% of the population report using recreational cannabis in the past three months, with 20.3% of males and 14% of females reporting such use [4].

While it is unclear how cannabis policies will evolve in other countries, there will undoubtedly be a rapidly evolving legal and social environment in various countries and jurisdictions, as cannabis use increasingly comes to the attention of regulators. However, its widespread use globally indicates that developing evidence of its impacts is already a critical global health issue. Hence, it is important to actively monitor recreational cannabis use patterns and trends, in particular in Canada and other jurisdictions where legalization has occurred, in order to understand the implications of such regulation and legalization.

It is clear from the wider substance use research field that sex- and gender-related factors (fully defined below) have a profound effect on substance use, the effects of use, and the response to interventions, approaches to treatment and overall policies [5]. As cannabis use trends evolve, it is therefore essential to collect and analyze evidence on sex and gender related factors and the effects on the benefits and risks of cannabis use. In the past, however, the integration of sex and gender concepts in substance use research and policy has often been overlooked [5], thereby preventing the building of evidence for effective programming for all sub populations and individuals. Integrating sex and gender in a disciplined manner within all future cannabis research will inform tailored harm reduction messaging, health information, and prevention and treatment responses for all genders.

### 1.1. How Do Sex and Gender Matter in Substance Use?

Sex related factors include the biological factors and mechanisms that are affected by, or affect, substance use in male and female bodies, while gender related factors include the effects on all people of gender norms, relations, identity and gendered institutional factors including customs, laws and regulations. Further, sex and gender related factors interact to influence patterns of substance use, effects of use, and responses to treatment. For example, Becker et al. argue that “gender and sex differences in addiction are a complicated interaction between sociocultural factors and neurobiological sex differences” [6]. It is also essential to take a transdisciplinary approach to addictions research in order to capture the myriad conceptual and theoretical perspectives that impact use and responses to substance use [7], including both sex and gender [8]. More specifically, investigating and analyzing interactions between aspects of sex and gender in cannabis research is an important step in understanding the full impact of cannabis use and legalization, as well as developing the required evidence for effective policy, programming, messaging and treatment.

**Figure 1:**
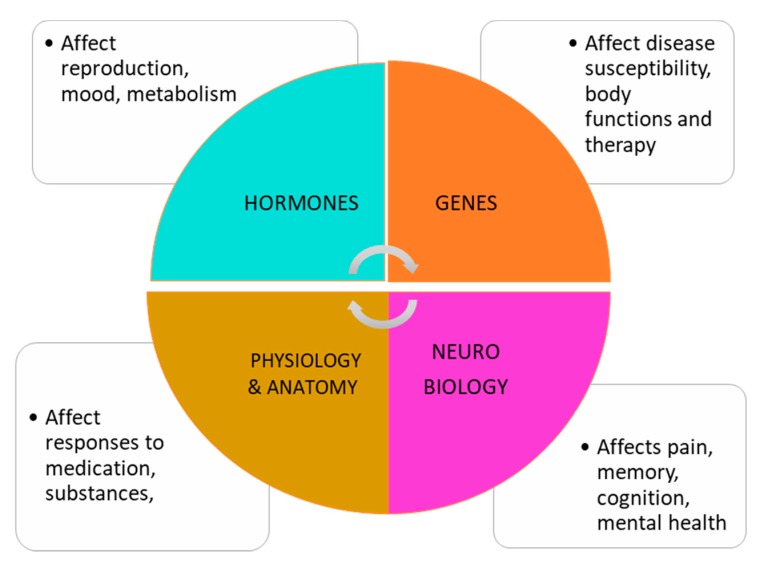
Sex related factors. These factors include reproductive characteristics, physiological processes, susceptibility to substances, and impacts on all body systems.

Sex related factors include a number of aspects of human biology, physiology, anatomy and genetics (see Figure 1). Bodily characteristics at birth are either male or female, with a small percentage of individuals who are labelled as intersex due to ambiguous characteristics [9]. These male or female characteristics contribute to a lifetime of developmental milestones, processes and stages, and determine reproductive capacity. In addition, a range of processes are affected by sex-based factors such as rates of metabolism, production of sex hormones, organ function, and development and distribution of adipose tissue, among others. These factors affect the ingestion of substances, including cannabis, and their rate of absorption, effects and impacts on the body and brain. These sex-based factors can also affect the response to therapeutic and treatment regimes, such as pharmaceutical treatments.

**Figure 2:**
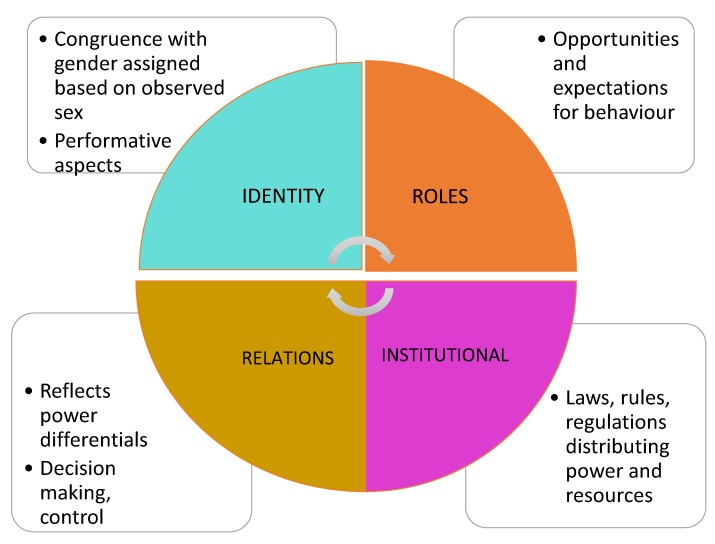
Gender related factors. These factors include culturally driven influences on relationships, opportunities, access to power, resources, decision making, autonomy, and identity.

Gender is often assumed or ascribed, based on our sex. Gender related factors are those connected to the gender relations we experience, the gender roles and norms to which we are exposed and influenced by, our gender identities (such as feminine, masculine, or gender diverse) and the gendered regulations and rules embedded in institutions such as education, politics and religion (see Figure 2). These factors are often temporal and culturally dependent and can change over time.

For example, substance use initiation patterns can be influenced by gendered relationships with partners or household members or friends, affecting our access and usage of cannabis. These influences can heighten dominant understandings of masculinity or femininity, and be expressed in intimate partnerships, peer groups, friendships, or families. Gendered roles such mothering or fathering are often impacted by substance use, in that caregiving is often seen as anathema to substance use, particularly for mothers for whom the stigma associated with substance use is acute. Gender identities and the ‘performance’ of our identities; whether feminine, masculine, or gender diverse (transgender, non-binary, or queer) have an impact on how and why substances such as cannabis are used, ingested, and in what contexts, not to mention how they are marketed and advertised when legal. Finally, large institutions affect substance use by imposing standards, moral teachings, or public education that implies restrictions, rules or opportunities based on gender, all of which can restrict or encourage substance use in gendered ways.

This wide range of gender related factors combine to create social and cultural contexts for substance use that in turn interact and intersect with the sex-based factors, along with a range of other characteristics such as sexual orientation, age, income, ethno/racial characteristics, Indigenous status, ability, rural and remote life, occupation, etc., to create both clinical and public health impacts.

In short, both sex and gender are of relevance and importance to any researcher or clinician investigating any substance use, including recreational cannabis. If such concepts are introduced (in disciplined and precise ways) into research designs, measured, analyzed, and reported, the resulting evidence will contribute to improved public education and health information for the population. This will enable the promotion of safe cannabis use, which is especially important in a rapidly changing policy environment. Further, including sex and gender in the analysis of the impact of policy can lead to more tailored and sharpened regulations, standards and public policy.

### 1.2. Gendered Trends of Use

Similar to most substances, more men than women use cannabis. According to data from the 2019 National Cannabis Survey in Canada, more men than women reported cannabis use in the past three months (20.3% vs. 14%). Further, men are more likely to report greater frequency of use and are twice as likely as women to report daily or almost daily use (8% of men vs. 4% of women). Indeed, after alcohol, cannabis is the most commonly used substance in Canada. These national statistics on cannabis use in Canada mirror evidence from the US and Europe where boys and men also report greater prevalence of cannabis use. Indeed, the prevalence of cannabis use among boys and men is higher compared to girls and women for past year [10,11,12], lifetime [13,14], and past 90 day use [15]. However, again, similar to historical trends of other substances there is evidence of a narrowing in the gender gap [14,16]. Some researchers suggest that the diffusion of cannabis use and experimentation appears similar to that observed with tobacco, with use beginning among men and more educated and higher income groups first, with later diffusion to women and lower socioeconomic status groups [16]. While population based data on prevalence of cannabis use among transgender populations are limited, a study conducted in the USA with *n* = 1210 transgender adults identified cannabis use among 24.4% of the sample; and cannabis use was significantly greater among transgender men compared to transgender women [17].

The majority of available studies and surveys have focused on gendered patterns and preferences for cannabis use. These forms of data are instrumental in offering insight into modes of use and trends of use. For example, there is evidence of gendered preferences for cannabis routes of administration [18], and that dominant gender norms may be reinforced or resisted through cannabis use behaviors [19]. With respect to sex related factors, some recent reviews have examined preclinical and clinical research on sex differences in the therapeutic effects of cannabis and potential for abuse [20] and on the sex-specific neurobiological mechanisms of cannabis use and dependence and associations with psychiatric symptoms [21]. Early evidence of sex differences from animal studies and (to a lesser extent) human studies suggests that females may be more sensitive to cannabis or cannabinoids in general [21], may transition to problematic cannabis use faster than males, and exhibit more intense withdrawal symptoms during abstinence [20].

Clearly, sex and gender related factors both matter in the context of cannabis use and there is much yet to explore in research and clinical practice. Overall, evidence on either sex or gender and cannabis use is lacking and nascent [22]. However, there is also a clear lack of research examining the interactions of sex and gender based factors on cannabis use and its effects. Hence, this paper draws on evidence from two scoping reviews to explore sex and gender based factors, and the potential interactions of these factors in the context of three key and current cannabis use practice and policy issues: cannabis use dependence, cannabis routes of administration (ROA), and driving under the influence of cannabis. These three exemplars comprise: a key clinical issue, a key health promotion/harm reduction issue, and key public policy issue. These are all important issues in early phases of legalized cannabis regimes, and all currently under scrutiny in Canada.

## 2. Materials and Methods

This article draws on evidence from two scoping reviews examining sex and gender related factors in the context of cannabis use, including: (1) a scoping review on sex, gender and substance use which identified *n* = 784 papers on cannabis; and (2) a more specific scoping review on sex, gender and cannabis routes of administration. The methods for the former are described in full in Hemsing and Greaves [23]. The methods for the latter review are described below.

### Scoping Review on Routes of Administration

We conducted a second scoping review of the academic and grey literature to identify literature on cannabis routes of administration (ROA). Specifically, we searched the literature for evidence on sex, gender and cannabis smoking or cannabis vaping, and health promotion, harm reduction and policy approaches to ROA.

The research questions were:

(Q1) How do sex and gender related factors impact:
  (a) The mode of cannabis or tobacco/nicotine use (ROA)?
  (b) The health effects of various cannabis routes of administration?
(Q2) What existing health promotion, harm reduction and policy approaches to cannabis ROA are available? Do these approaches include a sex/gender/equity lens?

The following academic databases were searched:

Medline, Embase (including Ovid), Cochrane; CINAHL, PsycINFO, Social Work Abstracts, Women’s Studies International, and Social Science Citation Index via Clarivate Analytics

The search covered studies published in the past 10 years (2009 to 2019) combining the following search terms on sex/gender and ROAs:

women; man; women; men; girl; boy; girls; boys; trans; transgender; female; male; sex; gender AND cigar*; e-cigar*; tobacco; nicotine; smoking; vaping; “heat not burn”; marijuana; cannabis; cannabinoid.

In total, *n* = 2332 studies were identified after duplicates were removed. One researcher screened abstracts and full papers, including studies that measured and analyzed some aspect of sex or gender, and that examined cannabis or tobacco routes of administration. Following abstract screening and full paper screening, *n* = 122 studies were included. In addition, we conducted a targeted search on the co-use of cannabis and tobacco and identified an additional 80 studies; after abstract and full paper screening, 19 of these studies were identified as relevant. In total, *n* = 131 papers were included in the scoping review.

This paper draws on some of the key findings from these two scoping reviews to consider the interplay of sex and gender in the context of cannabis use on three topic areas: cannabis use dependence, driving under the influence and cannabis routes of administration. Given the paucity of evidence on the interaction of sex and gender in cannabis use, relevant findings on sex, gender and substance use are included to explore biological and social interactions.

## 3. Results

### 3.1. Cannabis Use Dependence

Cannabis use disorder (CUD) affects approximately 10% of cannabis users [1]. It results in a range of symptoms, such as using cannabis in larger amounts or greater frequency than intended, challenges with cutting down, and withdrawal symptoms including anxiety and insomnia [24]. Despite the minority of users developing CUD, it is a high priority public health issue as dependency can interfere with social and economic activities, as well as negatively impact health and wellbeing. Monitoring the potential CUD effects post cannabis legalization is a key aspect of assessing the clinical impact of such legislation.

Sex and gender related factors both affect the development and impact of cannabis use disorder. Similar to other substance use, there is emerging evidence that females transition more quickly to cannabis use dependence compared to males, a process often called “telescoping” [25,26]. Cross-sectional studies analyzing US national data reported no differences between females and males in the age at first or heavy cannabis use, age at onset of CUD, total number of episodes of cannabis abuse or dependence, or in the number of criteria met for cannabis dependence [25]. However, the time from age at first use of cannabis to the age at onset of the CUD was longer among males (mean = 2.64 years vs. 2.24 years, F = 5.20, *p* < 0.05), providing support for telescoping among females who use cannabis [25].

Similarly, a second study found that while prevalence of CUD was greater among males, females reported a shorter duration from onset of cannabis use to onset of CUD compared to men (mean of 5.8 vs. 4.7 years) [26]. Clinical research also indicates greater abuse liability among females, such that females reported greater subjective effects at lower doses of oral THC (5 mg), while males reported greater subjective effects at higher doses (15 mg) [27]. The authors suggest these sex differences in subjective effects may contribute to the more rapid progression to dependence (telescoping) observed in females [27]. Clearly, more robust research on cannabis telescoping is needed to inform tailored prevention and harm reduction approaches for women and girls, in particular.

There is also emerging evidence that females may experience greater severity of cannabis dependence. In animal studies (mostly rodents) females have demonstrated slightly greater withdrawal symptoms compared to males, which is one component of dependence [28,29]. However, there are clear challenges in translating findings from animal to human studies including: more controlled experimental conditions, different methods of administration, the tendency to use synthetic forms of cannabinoids, and translational challenges due to the differences between animal and human bodies. Unfortunately, females tend to be underrepresented in human studies despite evidence of a telescoping effect [30].

However, there is some evidence based on self-reports of more severe CUD symptoms among women. Analysis of the US National Epidemiological Survey of Alcohol and Related Conditions (n = 43,093) found that both women and men who used cannabis reported a lower quality of life compared to those who did not use cannabis, and women and men with CUD reported a lower quality of life compared to those without CUD [31]. However, the negative effect of cannabis use on mental quality of life scores was more pronounced for women. Each daily joint smoked was associated with a greater decrease in mental quality of life summary scores in women compared to men [31]. This effect does not appear to be due to higher prevalence of depression among women who used cannabis, as there was no difference in the prevalence of mood and anxiety disorders between women and men in the sample who used cannabis. In our review, another study also reported greater CUD severity in women. In a sample of treatment-seeking adults with CUD, women reported greater withdrawal intensity, more co-occurring mental health issues (including lifetime panic disorder and current agoraphobia), and more days of poor physical health [32].

Both the telescoping effect and differences between women and men in the severity of CUD, may reflect the influence of sex hormones, the endocannabinoid system, and pharmacodynamics and pharmacokinetics. Neurobiological differences have been identified in the endogenous cannabinoid system of females and males. Studies examining the neural regions of rats have reported greater CB1 receptor desensitization and downregulation in females, which may in part explain cannabis telescoping among females [33]. In addition, sex hormones may modulate cannabinoid sensitivity [34,35,36]. However, studies on the influence of sex hormones on responses to cannabinoids in humans are lacking [21]. Cannabis pharmacodynamics and pharmacokinetics may also be implicated in the development of dependence. While sex differences in the metabolism of cannabinoids have been demonstrated in animal studies, these findings have not, to date, been found in human studies. For example, female rats metabolize THC more quickly than males [37,38], although this effect was reversed when CBD was provided to the rats before injection with THC [38]. Female rats also produce more 11-OH-Δ^9^-THC, the primary active metabolite of THC, while males metabolize THC to 11-OH-Δ^9^-THC and other inactive metabolites [21]. Further research is required to investigate the biological mechanisms underpinning sex differences in the progression to cannabis use dependence and the severity of cannabis use dependence.

While it has not yet been investigated, there may also be a gendered dimension to the greater severity of CUD reported by women. Women experience greater stigma and discrimination when they use substances of any kind, and this may partially explain the greater severity of CUD observed among women in some observational studies. For example, women may experience and report more shame and blame regarding their substance use, particularly if they are pregnant or parenting [39]. In general, women with substance use issues tend to experience more isolation and less social support compared to men [6]. The experience of stigma creates additional barriers to accessing substance use services and supports for substance use dependence and related health and social services, which may exacerbate the negative effects of women’s cannabis use dependence. Identifying the specific sex and gender factors associated with greater CUD severity among women, and tailoring treatment responses to ameliorate these risks is an important consideration for intervention development.

However, despite evidence of a telescoping effect and greater severity of CUD for females, males are more likely to be diagnosed with CUD [13] and tend to report a younger age of onset of CUD [40]. If there is evidence of a more rapid progression to dependence among females, and emerging evidence that females with CUD may be more severely impacted, why are men more likely to be diagnosed with CUD? As argued by Becker and colleagues, biological vulnerability does not equate to greater prevalence of dependence [6]. While biological factors impact the reinforcing effects of cannabis, social and environmental factors also influence the development of CUD [41]. Specifically, gender roles and norms may impact the risk of developing CUD. Men and boys tend to have greater prevalence of cannabis use, initiate earlier and use cannabis more frequently; and being male has been identified as one of the greatest risk factors for developing CUD [41].

Gender differences in the prevalence of substance use are in part due to men’s greater access to substances relative to women [42]. Substance use is more socially acceptable among boys and men relative to girls and women, and therefore men tend to have greater opportunities and access to substances in their social environments. In addition, adherence to dominant masculine norms boys has been associated with increased risk taking and substance use behaviors in general [43]. This is also reflected in the cannabis research literature suggesting that men tend to engage in riskier patterns of use, thereby increasing their risk of cannabis use dependence. For example, boys and men tend to report using a greater variety of cannabis routes of administration [18], and use higher potency cannabis products including cannabis concentrates [44] both of which increase the risk of dependence. With expanding cannabis legalization and increasing normalization of use, it will be crucial to monitor changes in gendered patterns of use and risks of dependence, particularly given the emerging evidence on greater biological vulnerability to dependence among females.

### 3.2. Routes of Administration

Routes of administration (ROA) refer to the various methods of using, inhaling or ingesting cannabis. These include: smoking, vaporizing, heating, ingesting oils or edibles, or using topical versions of cannabis such as creams. It is often assumed that smoking cannabis is the standard approach, both in popular culture and often implicitly, in policy and health promotion. However, examining and comparing the effects of ROAs using sex and gender related factors is essential to creating more precise and harm reducing health information and advice. The majority of the studies on cannabis ROAs that include an analysis of sex or gender have simply described prevalence and patterns of use. Men and boys tend to report higher rates of inhalation ROAs (smoking and vaping), including: joints, blunts, vaporizers, and concentrates [15,45], and water pipes/bongs [46]. There is some evidence that young women [47] and girls [48] may prefer edible cannabis products.

Several human studies have examined the pharmacokinetics of smoked cannabis. Some have demonstrated higher concentrations of THC and THC-COOH levels among females compared to males after administration of smoked [49,50] or vaporized cannabis [50], and greater subjective ratings of cannabis intoxication among females [49]. In contrast, a study with young adults aged 19–25 years who regularly used cannabis (1–4 days per week) found that females smoked less of the cannabis cigarette compared to males to reach their desired effect, but that blood THC and THC-COOH (a metabolite of THC) levels were lower among females compared to males even after adjusting for differences in the dose of THC inhaled [51]. The authors suggest that the similar subjective effects experienced by females at lower doses may reflect sex differences in the endocannabinoid system, as some animal studies have demonstrated greater cannabinoid type-1 (CB1) receptor availability and binding affinity with cannabinoids in females [51]. Ovarian hormones may also influence the subjective effects of cannabis in females; studies with other substances have revealed differences in subjective effects depending on menstrual cycle phase, though similar research on cannabis is currently lacking [51].

Further, Matheson et al. (2019) suggest that there may be sex differences in cannabis smoking topography [51]. In their experiment, females and males smoked for the same duration yet females smoked less of the cannabis cigarette suggesting they took smaller puffs, inhaled less deeply or held the smoke in the lungs for a shorter duration [51]. This finding may be influenced by neuro-biological factors, such as greater cannabinoid sensitivity among females [21], causing females to titrate their dose via their smoking behaviors. There could also be gender related influences; for example, a qualitative study with cannabis using women and men found that women often reported only smoking part of a joint, and typically avoided more “intense” ROAs such as water pipes/bongs, instead preferring a more gradual high [52]. The authors suggest these patterns of cannabis use align with feminine norms regarding the avoidance of excessive substance use and intoxication [52].

Recently, the emergence of e-cigarette or vaping associated lung injury (EVALI), highlights how gendered cannabis ROA preferences may shape health risks. EVALI has primarily affected young men (70%) in the USA and the majority of the reported cases have involved vaping THC products [53]. In the context of an unregulated market, young men may be more likely to access counterfeit cannabis vaping cartridges that are contaminated, increasing their risk of EVALI. Combined with broad improvements in the regulation of vaping products, tailored prevention and harm reduction responses are needed.

The preferences of women and girls for edible cannabis may reflect gender roles and norms regarding the social acceptability of substance use. Inhalation methods are more visible, while edible use can be easily concealed. This may be a more desirable option for girls and women, to avoid experiencing discrimination and stigma related to their cannabis use. This was reflected in a focus group study which found that girls reported a preference for edible cannabis because these products are more discreet [54]. However, given the challenges of titrating edible cannabis dosage, these trends and preferences signal the need for gender informed harm reduction messaging.

There is also evidence of differences in preferred ROA within groups of women and men. For example, women who are pregnant may prefer inhalation methods, because of the difficulty ingesting due to nausea [55]. This is an example of how biological factors—hormonal changes and/or pregnancy-related nausea, may underpin preferences for cannabis ROAs.

Culture can also intersect with gender roles to influence preferred routes of administration. Mixing cannabis with tobacco, often called “spliffs” is a common practice in some countries, particularly in the UK, European countries, and Australia [56]. In a qualitative study with Australian men who “mulled” (smoked a mixture of cannabis and tobacco), men described the effects of mixing tobacco and cannabis as producing a milder, more manageable “high” [56]. They described feeling more “grounded” than if they smoked only cannabis, and they preferred this effect as they were able to continue to participate in family and work responsibilities. In addition, blunt use–hollowed out cigars filled with cannabis, have been promoted through hip-hop culture [57], and are particularly popular among young Black males in the USA [58,59,60,61,62]. However, cannabis use ROAs that combine cannabis and tobacco, such as spliff and blunt use, confer greater risk of dependence [57] as well as adverse respiratory health effects. Gendered and/or culturally sensitive harm reduction messaging that addresses the risks associated with co-use of cannabis and tobacco is warranted.

In short, the sex and gender interactivity affecting ROA choices and effects should be areas of key concern to clinicians, researchers, and health promotion and harm reduction specialists. Currently precise and gendered health information aimed at the general public about ROA choices is lacking, along with tailored information that includes basic evidence on sex and gender.

### 3.3. Driving Under the Influence of Cannabis

Driving under the influence of cannabis is a key public policy issue in jurisdictions that have legalized recreational cannabis. Discussions about legalization of cannabis often focus on estimating risks associated with possible impaired driving. Not surprisingly, after the legalization of cannabis in Canada in 2018 there has been increased interest in understanding cannabis related impairment and preventing and responding to driving under the influence of cannabis. This emphasis formed one of the key focal areas of health promotion and messaging campaigns aimed at young people in particular [63].

Impaired driving is a gendered activity, with the prevalence of driving after cannabis use higher among men. In a Swedish study, a greater proportion of men were apprehended with THC concentrations detected in their blood (94% vs. 6%), and among those with detected THC, blood concentrations were higher in men than in women (mean 2.1 ng/mL vs. mean 1.4 ng/mL) when cannabis was the only substance detected [64]. In a US study, among college students who reported past month cannabis use, 43.9% of males and 8.7% of females reported driving after cannabis use [65]. In addition, males were more likely to report riding as a passenger with someone who had recently used cannabis (51.2% vs. 34.8%). O’Malley and colleagues’ analysis of US high school seniors also found that male students were more likely to report driving after smoking cannabis; however, there was no gender difference in riding as a passenger after cannabis use [66].

Gendered patterns of cannabis use likely influence the risk of driving under the influence of cannabis. In one study, males were more likely to both vape and use cannabis edibles; and more frequent vaping was associated with driving under the influence [67]. As discussed above, in general, boys and young men tend to engage in riskier substance use behaviors. Boys and men are also more likely to co-use cannabis and alcohol, which significantly increases impairment, driving errors and accidents [68]. Further, compared to women, men tend to perceive lower harm with driving under the influence of cannabis, are less likely to believe that cannabis negatively affects their driving ability, are more likely to perceive their friends as approving of driving under the influence of cannabis, and are more likely to report an intention to drive after cannabis use in the future [69]. These gendered patterns of cannabis use and beliefs and perceptions are clear and critically important targets for gender specific harm reduction and health promotion efforts. Specifically, gender informed harm reduction messaging is needed that addresses both driving after cannabis use and riding as a passenger with a driver who has recently consumed cannabis.

Sex differences in the subjective effects of cannabis may impact impairment. As noted above, the greater subjective effects that females tend to experience at lower doses and with a lower blood level of THC may suggest the potential for greater impairment with a lower dose of cannabis among females [51]. It is possible that females may require more time to achieve sobriety before driving, though further research is required to investigate sex differences in the metabolism of cannabinoids and the effects on intoxication and impaired driving. This evidence can be used to inform more precise and refined harm reduction and health promotion responses, messages, and recommendations. Sex specific measures of impairment are lacking; further research is needed to understand sex differences in cannabis impairment and effects on attention and driving to inform measures of impairment and enforcement of impaired driving laws.

It is clear that more research is needed to examine sex differences in driving related impairment. In simulated studies by Anderson et al., they found no evidence of any sex differences. They studied the effects of inhaled THC on attention impairment among people who used cannabis occasionally (participants reported using cannabis at least once per month, but no more than 10 times per month), while driving in a simulator [70], and found no sex differences in the impact of cannabis use. In another study, participants reduced their overall driving speed and performed more poorly on a neuropsychological test following the driving simulation, but no sex differences were observed [71]. In short, this vital area of public policy is still lacking in research that would enable health promoters and enforcement officials to better target their messaging and policy using comprehensive sex and gender related evidence.

## 4. Discussion

Research on sex and gender related factors and cannabis use and its effects is in its infancy. This area needs considerable attention and growth in light of the high level of cannabis use globally, as well as the legalization of cannabis in various jurisdictions. We have reported elsewhere on known sex and gender related factors that appear to affect use, impact and effect of cannabis use [22]. In this article we have elucidated the various components of sex and gender that are relevant to the study of recreational cannabis use (and other substances) and illustrated how sex and gender interact and combine their effects. We illustrated these interactions in three examples relevant to health outcomes, health promotion and public policy: the development of CUD, the differential choices and impacts of ROA, and cannabis impaired driving.

Aside from one review that acknowledges the influence of both social and biological factors on cannabis use [41], most of the literature we found in our searches for sex and gender influences on cannabis use and ROA has examined either sex related or gender related factors. Going forward, a framework may be useful for examining the interactions of sex and gender, along with other social dimensions of health and equity. There have been calls for understanding intersectional factors affecting health, including the intersection of sex and gender [72]. While intersectional frameworks have been criticized for not adequately attending to biological factors, some proponents have identified opportunities for integrating biological and social dimensions of health within this framework, and begun to consider how biological factors intersect with other factors including gender, class, and ethnicity to address health inequities [73].

Physiological aspects of sex are increasingly understood as being influenced by gender-related social dynamics [74]. Yet, most of the evidence on gender and cannabis to date has focused on noting simple differences between women and men and boys and girls in patterns and prevalence of use. The evidence on sex differences in cannabis use is largely confined to animal studies, and studies on humans have not consistently included female participants and/or integrated a full sex-based analysis. More research is needed to understand how male and female bodies respond to cannabis use and the respective health consequences of use, and the influence of social factors on biological mechanisms. This evidence can then be used to inform more precise harm reduction and health promotion messaging, similar to the sex specific Canadian Lower Risk Drinking Guidelines [75]. Overall, the development of precise, sex, and gender tailored responses to cannabis use are needed to reduce harms, maximize benefits, and improve clinical treatment and health promotion.

Despite these current limitations and insufficiencies, these early findings on cannabis use dependence, cannabis routes of administration, and driving while under the influence have important implications for prevention, health literacy, public education, and treatment. For example, tailored messaging is needed to address risky patterns and consequences of use among boys and men, including greater and more frequent cannabis use, inhaling high potency/high THC products, co-use with alcohol and tobacco, and driving or riding with drivers under the influence of cannabis. For women, emerging evidence on female vulnerabilities to developing dependence and severity of CUD could inform prevention and treatment responses. If telescoping occurs more quickly in females, compounded by increased social stigma directed at women, treatment options should be more readily available for women at the earliest stage possible.

In addition, gender specific efforts can be made to address and reduce discrimination and stigma for all groups, via public education and in the design and delivery of substance use services. Further, considering sex and gender together in cannabis use, can pave the way for gender transformative initiatives in health promotion and messaging. Such approaches simultaneously reduce risky use and work toward gender and health equity in cannabis prevention, harm reduction and treatment responses, thereby alleviating inequities associated with cannabis use [76,77].

It will be critical to continue to monitor and collect data on gendered cannabis use patterns. Patterns of use may change as recreational cannabis becomes increasingly normalized, and producers and advertisers tailor product promotions to target specific groups. As discussed, the gender gap appears to be narrowing [16] and there are indications that cannabis vapour product producers are marketing specific devices to girls and women [78]. If the gender gap in cannabis use continues to narrow, and girls and women begin to use different cannabis ROAs, this will likely affect their health and social consequences of cannabis use including cannabis dependence and driving under its influence.

## 5. Conclusions

While research on recreational cannabis use is rapidly expanding in response to a shifting policy landscape, research specifically focussed on the impact of sex and gender on its use is in its infancy. More adherence and precision is required in applying sex and gender related concepts to the study of substance use in general, and cannabis use in particular. Robust studies are needed to investigate a full spectrum of sex related factors in the effects of cannabis use; and to explore how gender norms, roles, relations and identities all impact cannabis use and health and social consequences. Further, as illustrated using the examples of CUD, ROA and impaired driving, research is needed that examines the interactions of sex with gender related factors and other social determinants of health including class, age, income, and ethnicity to address and prevent inequities in health related to cannabis use. Advancing knowledge on the interaction of sex, gender and equity based factors will inform more responsive health promotion, effective harm reduction, and precise treatment approaches for all genders.
